# Early sexual debut and pregnancy termination: uncovering the link among sexually active young women in 23 sub-Saharan African countries

**DOI:** 10.1186/s40834-024-00323-6

**Published:** 2024-11-18

**Authors:** Obasanjo Afolabi Bolarinwa

**Affiliations:** 1 Department of Public Health, York St John University, London, UK; 2 Department of Demography and Population Studies, University of Witwatersrand, Johannesburg, South Africa

**Keywords:** Age at sexual debut, Sexually active, Young women, Pregnancy termination, Sub-Saharan Africa

## Abstract

**Background:**

Unplanned pregnancy could be a socio-economic burden for many young women in sub-Saharan Africa (SSA) which often leads to pregnancy termination. The role of age at sexual debut in pregnancy termination in countries with lower income remains unknown. Hence, this study examines the association between age at sexual debut and pregnancy termination among sexually active young women between the ages of 15 -24 in 23 SSA countries.

**Methods:**

Cross-sectional secondary datasets from the most recent Demographic and Health Survey conducted in 23 countries in SSA conducted between 2010 and 2018 among 34,343 sexually active young women were analysed using bivariate and multivariable logistic regression to examine the association between age at sexual debut and pregnancy termination with statistical significance of p < 0.05.

**Results:**

The pooled prevalence of pregnancy termination among sexually active young women in SSA was 11.00%. Higher odds of pregnancy termination were found among those who had early sexual debut (below 16 years) [(aOR = 1.34, 95% CI = (1.22–1.48)] compared to those who had late sexual debut. Furthermore, those who were exposed to mass media [(aOR = 1.29, 95% CI = (1.16–1.43)] were more likely to report pregnancy termination compared to those who were not exposed. On the other hand, those residing in rural areas and those within the richest wealth index were associated with lower odds of pregnancy termination.

**Conclusion:**

The study concluded that early sexual debut of sexually active young women in SSA was significantly associated with pregnancy termination. Mass media exposure was found to be a risk factor for pregnancy termination whilst residing in rural areas, and those within the richest wealth index were protective factors. Interventions should be designed to target young women with early sexual debut to provide comprehensive sexual and reproductive health education to enable them to make informed decisions on pregnancy termination.

## Background

Pregnancy termination among young women continues to be a major contributor to adverse health outcomes and socio-economic burden for many young women and their families globally and, most especially, in sub-Saharan African countries [1–4]. Approximately 40 million out of 80 million unplanned and mistimed pregnancies that occurred in low-and-middle-income countries (LMICs) ended as pregnancy termination [1, 3].

Most sub-Saharan African countries have restrictions on pregnancy termination services, with no liberal laws permitting pregnancy termination [5]. For instance, in Nigeria, the criminal code criminalised abortion, while in Ethiopia, the revised penal code only allows abortion in cases of rape, incest or fetal impairment [6]. As of 2019, it is estimated that around 6.2 million unsafe abortions occur annually in sub-Saharan Africa (SSA). This region still has the highest rate of abortion-related deaths globally, with approximately 185 maternal deaths per 100,000 abortions [7]. However, more than 75% of these terminations were classified as unsafe due to the engagement of untrained medical practitioners and/or non-recommended methods of terminating the pregnancies, which could lead to adverse health outcomes such as infections and sometimes fatality [2, 4, 8]. In the same vein, unsafe pregnancy termination is the leading cause of maternal mortality among young women aged 15–24 years in this region [9, 10].

Frequent sexual activities and non-compliance to contraceptive use regimens have been cited in several studies as major factors exacerbating pregnancy termination [1, 10, 11]. These two factors are more prevalent among young women, hence their significant contribution to the regional burden of pregnancy termination of 57% among young women aged 15–24 years in SSA [12]. Other factors that have been identified as contributors to the continuous pregnancy termination demands among young women include parity, place of residence, region of residence, working status, marital status, etc. [13, 14].

A 2015 study in the United States found that nearly 70% of young women had their first sexual intercourse by age 19 and that the onset of sexual activity among young women is regarded as a normative stage where decisions relating to sexual and reproductive health are crucial [15]. In this regard, early sexual debut without commiserating the right reproductive health information could expose the group to higher poor reproductive health decision-making, which could lead to adverse reproductive health outcomes such as unintended pregnancy [16, 17] and a host of other adverse sexual health outcomes like multiple sexual partners, sexually transmitted infections, frequent sexual activities with risky partners [18–21].

As pregnancy termination continues to be a major global health challenge, a plethora of studies have linked several demographic and economic factors to pregnancy termination at country and regional levels [1, 2, 13, 14, 22, 23]. However, the significant link between age at sexual debut and pregnancy termination among sexually active young women has not received the required attention in the literature. Thus, this study addresses the literature gap by examining the association between age at sexual debut and pregnancy termination among sexually active young women in SSA.

The potential outcome of this study is important because it will provide the information needed to design specific sexual and reproductive health interventions to reduce unwanted and unsafe pregnancy termination among sexually active young women in SSA, and this is the first study that pooled the latest dataset together across SSA to examine the time of sexual debut as an important link to pregnancy termination among sexually active young women in SSA.

## Materials and methods

### Study design and data source

This study involved a cross-sectional analysis of Demographic Health Survey (DHS) datasets from 23 sub-Saharan African countries conducted between 2010 and 2018. DHS is a nationally representative dataset that has been conducted in more than eighty-five LMICs. The survey data collection is done using different questionnaires to collect information from respondents on several health indicators relating to sexual and reproductive health, including maternal and child health, family planning, fertility, and gender-based violence [24].

A two-stage cluster random sampling technique was employed to sample respondents for the surveys. In the first stage, clusters (such as villages or urban areas) were selected. In the second stage, households within the selected clusters were sampled. After administering the household schedule, eligible women were identified for individual interviews [25]. The full details of the sampling processes used in the DHS are well documented by Aliaga and Ruilin [25].

This study sample was drawn from the individual women’s recode files, which were derived from individual women’s questionnaires from all the countries included. A total of 34,343 sexually active young women aged 15–24 with complete cases on variables of interest were included in the final analysis. The inclusion criteria were young women who were sexually active and had responses for “age at sexual debut” and “pregnancy termination” responses. Young women who were not sexually active and who did not have a response for “age at sexual debut” and “pregnancy termination” were excluded or dropped from the study analysis. Young women were deemed not sexually active if the response was “never had sex” and/or not active in the last 4 weeks” [25].

The study relied on and utilised the strengthening of the reporting of observational studies in epidemiology (STROBE) statement in developing the study’s manuscript [26]. Sample size distribution by country is presented in Table 1 below. The datasets for the DHS can be accessed freely for all countries included in this study at https://dhsprogram.com/data/available-datasets.cfm.

**Table 1:** Sample size distribution by country

Survey countries	Survey year	Weighted sample	Percentage
**Central Africa**			
Angola	2016	1427	4.16
Cameroon	2019	983	2.86
Chad	2015	1302	3.79
**West Africa**			
Benin	2018	2192	6.38
Burkina Faso	2010	1519	4.42
Cote d'Ivoire	2012	444	1.29
Ghana	2014	1415	4.12
Guinea	2018	490	1.43
Liberia	2013	1697	4.94
Mali	2018	1950	5.68
Niger	2013	1807	5.26
Nigeria	2018	1131	3.29
Senegal	2018	741	2.16
Sierra Leone	2013	2511	7.31
Togo	2014	1647	4.80
**East Africa**			
Ethiopia	2016	844	2.46
Kenya	2014	724	2.11
Tanzania	2016	658	1.92
Uganda	2011	2478	7.22
**Southern Africa**			
Comoros	2012	1810	5.27
Lesotho	2014	517	1.50
Mozambique	2011	4887	14.23
Zimbabwe	2018	1166	3.40

### Study variables

#### Outcome variable

The study’s outcome variable is pregnancy termination. DHS included miscarriages, stillbirths, and induced abortions as pregnancy termination. The respondents were asked if they had ever had pregnancy termination to derive this variable. The responses were documented in the binary form “no” and “yes”. Sexually active young women who never had a pregnancy termination in the past were coded “0,” representing “no,” while those who have had pregnancy termination were coded “1” representing “yes”. Similar coding has been used in several studies conducted in the past [1, 8].

#### Key explanatory variable

The main explanatory variable is the age at sexual debut. DHS asked questions on the age the respondents had their sexual debut. Those who had their first sex at age 16 years and above were coded as “0,” while those who had their sexual debut below 16 years were coded as “1” [27, 28].

#### Covariates

A total of ten (10) relevant covariates were included in this study. These variables include age, place of residence, educational level, partner’s educational level, working status, wealth index, parity, unmet needs for contraception, knowledge of any method of family planning, and mass media exposure. These variables were not determined a priori; instead, they were based on previous significant associations with pregnancy termination [29, 30]. Except for the place of residence (rural/urban), working status (No or Yes), and wealth index (poorest, poorer, middle, richer, richest), where the existing DHS coding was maintained, the remaining covariates were recoded. The other covariates and their recoding include age (15–19, 20–24) to conform with the study objective of young women only; educational level (no education, primary, secondary education and above); partner’s educational level (no education, primary, secondary education and above); parity (0, 1–3, 4 and above); unmet needs for contraception (no unmet need, unmet need); knowledge of any family planning method (knows no method, knows method); and lastly mass media exposure (No exposure, had exposure). Mass media exposure was regarded as respondents listening to the radio, watching television, and reading newspapers/magazines. Those without this exposure were coded as “no exposure," while those exposed to at least one media medium were coded as “had exposure” [29, 31, 32]. It is important to know that the rationale for excluding women above 24 years was based on their “age reported,” i.e. the selection of young women (age 15 to 24) was not based on age of sexual debut but the age of respondents [27].

### Statistical analyses

Data extraction, cleaning, recoding, and analyses were carried out using Stata software version 17.0. A forest plot was used to show the prevalence of both pregnancy termination and age at sexual debut among sexually active women in SSA by country. Next was the age distribution at sexual debut, selected covariates across pregnancy termination, while the Pearson chi-square test (χ^2^) was conducted to determine the association between age at sexual debut, selected covariates, and pregnancy termination (Table 2).

**Table 2:** Distribution of age at sexual debut, selected covariates across pregnancy termination among sexually active young women in sub-Saharan Africa (*N* = 34,343)

Variables	Frequency (n)	Percentage (%)	Pregnancy termination		*p*-value (χ^2^)
Key independent			No	Yes	
**Age at sexual debut**					*p* < 0.001
16 years & above	22,326	65.01	90.08	9.92	
Below 16 years	12,017	34.99	88.31	11.69	
Covariates					
**Age**					*p* < 0.001
15–19	10,364	30.18	91.91	8.09	
20–24	23,979	69.82	88.40	11.60	
**Place of residence**					*p* < 0.001
Urban	9,924	28.90	87.57	12.43	
Rural	24,419	71.10	90.23	9.77	
**Educational level**					*p* < 0.001
No education	14,090	41.03	91.17	8.83	
Primary education	10,834	31.55	88.40	11.60	
Secondary & above	9,419	27.43	88.12	11.88	
**Partner’s educational level**					*p* < 0.001
No education	12,741	37.10	91.48	8.51	
Primary education	9,730	28.33	89.15	10.85	
Secondary & above	11,871	34.57	87.54	12.46	
**Working status**					*p* < 0.001
No	17,114	49.83	90.55	9.45	
Yes	17,229	50.17	88.38	11.62	
**Wealth index**					*p* < 0.01
Poorest	7,308	21.28	90.05	9.95	
Poorer	7,754	22.58	90.33	9.67	
Middle	7,055	20.54	89.10	10.90	
Richer	6,781	19.74	89.54	10.46	
Richest	5,446	15.86	87.80	12.20	
**Parity**					*p* < 0.01
No child	7,477	21.77	88.37	11.63	
1–3 Children	25,264	73.56	89.66	10.34	
4 & above	1,602	4.66	91.47	8.53	
**Unmet needs for contraception**					*p* < 0.001
No unmet need	26,719	77.80	88.97	11.03	
Unmet need	7,624	22.20	91.19	8.81	
**Knowledge of any FP method**					
Knows no method	2,767	8.06	94.99	5.01	
Knows method	31,576	91.94	88.98	11.02	
**Mass media exposure**					*p* < 0.001
No exposure	11,999	34.94	91.58	8.42	
Had exposure	22,344	65.06	88.32	11.68	

Weighted DHS

*FP* family planning

Two regression models (Model I & Model II) were fitted to determine the association of age at sexual debut, selected covariates, and pregnancy termination in the selected SSA countries. The first model (model I) examined the association of age at sexual debut on pregnancy termination as a univariate regression model (Bivariate), while the second model (model II) was used to determine the adjusted association between age at sexual debut, selected covariates and pregnancy termination using a multivariable regression model. The results of the regression analyses were presented in a tabular form using crude odds ratio (cOR) representing model I and adjusted odds ratio (aOR) representing model II with their respective 95% confidence interval (CIs), as presented in Table 3.

**Table 3:** Bivariate and multivariable of age at sexual debut, selected covariates, and pregnancy termination among women in sub-Saharan Africa (*N* = 34,343)

Variable	Unadjusted (Model I)	Adjusted (Model II)
Key independent	cOR[95% CI]	aOR[95% CI]
**Age at sexual debut**		
16 years & above	1	1
Below 16 years	1.20***[1.10–1.31]	1.34***[1.22–1.48]
Covariates		
**Age**		
15–19	1	1
20–24	1.49***[1.35–1.64]	1.65***[1.47–1.84]
**Place of residence**		
Urban	1	1
Rural	0.76***[0.69–0.84]	0.85*[0.75–0.97]
**Educational level**		
No education	1	1
Primary education	1.35***[1.22–1.50]	1.13[1.00–1.28]
Secondary & above	1.39***[1.25–1.55]	0.01[0.87–1.17]
**Partner’s educational level**		
No education	1	1
Primary education	1.31***[1.17–1.46]	1.14*[1.00–1.30]
Secondary & above	1.53***[1.38–1.69]	1.30***[1.14–1.49]
**Working status**		
No	1	1
Yes	1.26***[1.15–1.38]	1.17**[1.07–1.28]
**Wealth index**		
Poorest	1	1
Poorer	0.97[0.85–1.10]	0.91[0.80–1.03]
Middle	1.11[0.97–1.26]	0.95[0.83–1.08]
Richer	1.06[0.92–1.21]	0.81**[0.69–0.94]
Richest	1.26**[1.09–1.44]	0.82*[0.68–0.99]
** Parity**		
No child	1	1
1–3 Children	0.88*[0.79–0.97]	0.70***[0.62–0.78]
4 & above	0.71**[0.56–0.89]	0.52***[0.40–0.66]
**Unmet needs for contraception**		
No unmet need	1	1
Unmet need	0.78***[0.70–0.87]	0.78***[0.70–0.87]
**Knowledge of any FP method**		
Knows no method	1	1
Knows method	2.35***[1.91–2.88]	1.86***[1.50–2.31]
**Mass media exposure**		
No exposure	1	1
Had exposure	1.44***[1.31–1.58]	1.29***[1.16–1.43]
**Survey’s year**		
2010	1	1
2011	1.22[0.98–1.51]	1.16[0.94–1.45]
2012	1.07[0.84–1.36]	1.21[0.94–1.55]
2013	0.82[0.65–1.05]	1.09[0.85–1.40]
2014	1.13[0.90–1.42]	0.95[0.75–1.20]
2015	1.26[0.95–1.68]	1.21[0.91–1.60]
2016	1.67[0.90–1.51]	1.13[0.87–1.46]
2018	0.94[0.76–1.18]	0.94[0.75–1.18]
2019	1.95***[1.45–2.64]	2.03***[1.50–2.74]

Model I: unadjusted model examining age at sexual debut and selected covariates on pregnancy termination; Model II: adjusted the selected covariates as confounders; AOR is the adjusted odds ratio, COR is the unadjusted odds ratio; Exponentiated coefficients; 95% CI is the 95% confidence intervals

*p < 0.05

**p < 0.01

***p < 0.001

The statistical model employed a logistic regression model to estimate the odds of pregnancy termination as a function of age at sexual debut and other covariates. The general form of the model was expressed as;

$$\text{log}\left(\frac{P\left(pregnancy termination\right)}{1-P\left(pregnancy termination\right)}\right)={\beta }_{0}+{\beta }_{1} \times age at sexual debut+{\beta }_{2 }\times control variables$$

While the goodness of fit was tested using the Homer-Lemeshow Test.

Statistical significance was set at a p-value less than 0.05. A weight of (v005/1000000) was applied during the statistical analysis to avoid oversampling and non-response errors. The survey Stata command “svy” was used to adjust to the complex sampling structure of the DHS data in the chi-square and regression analyses. The multicollinearity test, which used the variance inflation factor (VIF), revealed no evidence of collinearity amongst the independent variables and covariates. All the analyses were carried out using Stata version 17.0 (Stata Corporation, College Station, TX, USA).

## Results

### Prevalence of pregnancy termination among sexually active young women in SSA

**Fig. 1:**
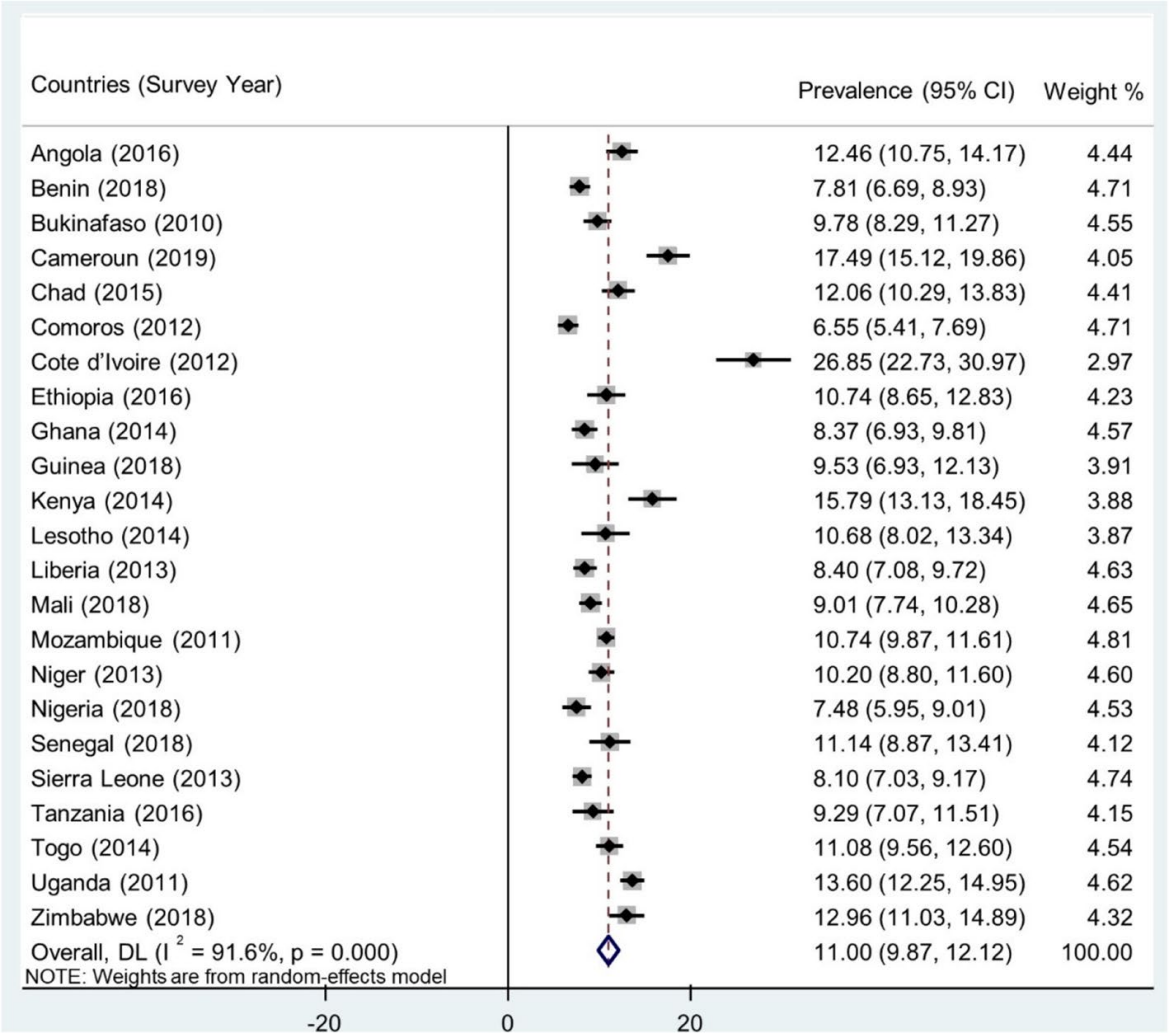
Forest plot showing the prevalence of pregnancy termination among sexually active young women in sub-Saharan Africa by country

In the 23 countries in SSA, the pooled prevalence of pregnancy termination among sexually active young women was 11.00% (95% CI 9.87% to 12.12), ranging from as high as 26.85% (95% CI 22.73% to 30.97%) in Cote d'Ivoire to as low as 6.55% in Comoros (95% CI 5.41% to 7.69%) (Fig. 1).

### Prevalence of age at sexual debut among sexually active young women in sub-Saharan Africa

**Fig. 2:**
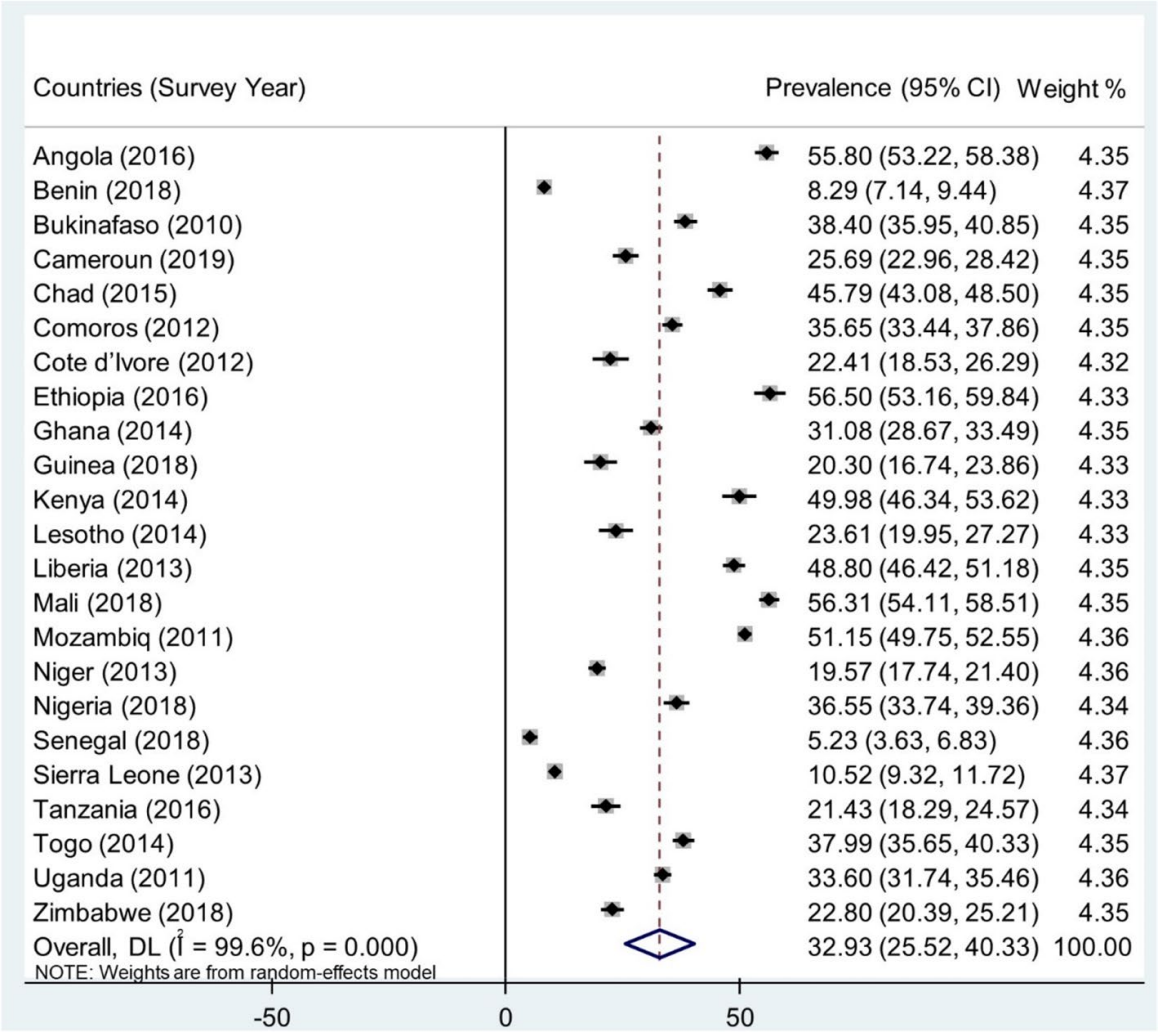
Forest plot showing the prevalence of age at sexual debut among sexually active young women in sub-Saharan Africa by country

In the 23 countries in SSA, the pooled prevalence of age at sexual debut among sexually active young women was 32.93% (95% CI 25.52% to 40.33), ranging from as high as 56.50% (95% CI 53.16% to 59.84%) in Ethiopia to as low as 5.23% in Comoros (95% CI 3.631% to 6.83%) (Fig. 2).

### Distribution of age at sexual debut, covariates across pregnancy termination among sexually active young women in sub-Saharan Africa

Table 2 shows the age distribution at sexual debut and selected covariates across pregnancy termination among 34,343 sexually active young women in SSA. The results show that 90.08% of sexually young women who had a sexual debut from 16 years and above reported no pregnancy termination, while 11.69% of those who had a sexual debut below the age of 16 years have had pregnancy termination. In SSA, 11.6% of sexually active young women aged 20–24 have previously experienced a pregnancy termination. Sexually active young women and their partners with secondary education and above in SSA had 11.88% and 12.46% pregnancy termination, respectively. 11.68% of respondents exposed to mass media reported having had pregnancy termination. The chi-square (χ^2^) results show that age at sexual debut and all the selected covariates were significantly associated with pregnancy termination.

### Association of age at sexual debut, covariates, and pregnancy termination among sexually active young women in sub-Saharan Africa

Two models were fitted to examine the association between age at sexual debut, covariates and pregnancy termination among sexually active young women in SSA, and the results are presented in Table 3. The variables that were significant to pregnancy termination among sexually active young women in SSA include age at sexual debut, age of young women, place of residence, partner’s educational level, working status, wealth index, parity, knowledge of family planning method, unmet needs for contraception, mass media exposure, and year of study.

The adjusted model (Model II) revealed that sexually active young women in who initiated sexual activity before age 16 had 34% higher odds of experiencing pregnancy termination (aOR = 1.34, 95% CI = 1.22–1.48) compared to those whose sexual debut was at age 16 or older.

Additionally, the analysis of covariates showed that women aged 20–24 (aOR = 1.65, 95% CI = 1.47–1.84), those with partners having secondary education or higher (aOR = 1.30, 95% CI = 1.14–1.49), women who were employed (aOR = 1.17, 95% CI = 1.07–1.28), and those knowledgeable about family planning (aOR = 1.86, 95% CI = 1.50–2.31) had significantly higher odds of pregnancy termination. Furthermore, mass media exposure (aOR = 1.29, 95% CI = 1.16–1.43) and surveys conducted in 2019 (aOR = 2.03, 95% CI = 1.50–2.74) also increased the likelihood of pregnancy termination compared to reference groups.

On the other hand, sexually active young women in SSA who reside in rural areas [(aOR = 0.85, 95% CI = (0.95–0.97)], those within the richest wealth index [(aOR = 0.82, 95% CI = (0.68–0.99)], young women with 4 children and above [(aOR = 0.52, 95% CI = (0.40–0.66)], and respondents with unmet need for contraception [(aOR = 0.78, 95% CI = (0.70–0.87)] had a lower likelihood of reporting pregnancy termination compared to sexually active young women residing in the urban area, those from poorest wealth index, young women with no child, and those with no unmet need for contraception.

### Association between age at sexual debut and pregnancy termination among sexually active young women in sub-Saharan Africa by sub-region/country

In Model II, a higher likelihood of pregnancy termination among sexually active young women in SSA who had sexual debut below age 16 years were reported in three sub-regions out of four sub-regions included in the study and these are West Africa [aOR = 1.23; 95%(CI = 1.01–1.38)], East Africa [aOR = 1.31; 95%(CI = 1.10–1.57)], and Southern Africa [aOR = 1.54; 95%(CI = 1.31–1.82)] while higher likelihood of sexual debut below age 16 years and pregnancy termination were reported in countries like Chad [aOR = 1.49; 95%(CI = 1.04–2.13)], Cote d'Ivoire [aOR = 2.55; 95%(CI = 1.46–4.47)], Liberia [aOR = 1.64; 95%(CI = 1.11–2.43)], Mali [aOR = 1.46; 95%(CI = 1.05–2.04)], and Mozambique [aOR = 1.55; 95%(CI = 1.26–1.91)] (Table 4).

**Table 4:** Bivariate and multivariable analysis of age of sexual debut and pregnancy termination among young women in sub-Saharan Africa (*N* = 34,343)

**Sub-regions** Countries (Survey year)	**Model I** **cOR [95% CI)**	**Model II** **aOR [95% CI]**
**Central Africa**	**0.91[0.75–1.11]**	**1.15[0.93–1.42]**
Angola (2016)	0.67*[0.47–0.96]	0.92[0.62–1.35]
Cameroon (2019)	1.30[0.90–1.89]	1.38[0.92–2.06]
Chad (2015)	1.24[0.90–1.71]	1.49*[1.04–2.13]
**West Africa**	1.19**[1.06–1.33]	1.23***[1.10–1.38]
Benin (2018)	1.47[0.90–2.38]	1.59[0.97–2.61]
Burkina Faso (2010)	1.02[0.72–1.45]	1.30[1.00–4.54]
Cote d'Ivoire (2012)	1.89*[1.16–3.07]	2.55**[1.46–4.47]
Ghana (2014)	1.19[0.78–1.81]	1.22[0.78–1.90]
Guinea (2018)	1.09[0.53–2.24]	1.35[0.60–3.01]
Liberia (2013)	1.39[0.98–1.99]	1.64*[1.11–2.43]
Mali (2018)	1.11[0.81–1.51]	1.46*[1.05–2.04]
Niger (2013)	0.83[0.54–1.28]	1.04[0.67–1.64]
Nigeria (2018)	0.97[0.60–1.57]	0.97[0.59–1.59]
Senegal (2018)	0.74[0.29–1.91]	0.79[0.29–2.13]
Sierra Leone (2013)	1.49[0.96–2.30]	1.28[0.81–2.00]
Togo (2014)	1.26[0.92–1.73]	1.31[0.93–1.84]
**East Africa**	**1.16[0.98–1.38]**	1.31**[1.10–1.57]
Ethiopia (2016)	1.16[0.75–1.79]	1.38[0.84–2.25]
Kenya (2014)	1.06[0.72–1.56]	1.10[0.74–1.63]
Tanzania (2016)	1.32[0.68–2.54]	1.73[0.84–3.56]
Uganda (2011)	1.11[0.88–1.40]	1.23[0.96–1.57]
**Southern Africa**	1.31***[1.13–1.51]	1.54***[1.31–1.82]
Comoros (2012)	1.38[0.93–2.04]	1.41[0.92–2.15]
Lesotho (2014)	1.76[0.94–3.28]	1.84[0.92–3.67]
Mozambique (2011)	1.27*[1.05–1.53]	1.55***[1.26–1.91]
Zimbabwe (2018)	1.16[0.75–1.79]	1.32[0.81–2.14]

Model I: unadjusted model examining the age at sexual debut and pregnancy termination with selected confounders separately; Model II: adjusted for the selected covariates (age, place of residence, educational level, partner’s educational level, working status, wealth index, parity, unmet needs for contraception, knowledge of any FP method, and mass media exposure) as confounders; AOR is the adjusted odds ratio, COR is the unadjusted odds ratio; sub-region results in bold

*p < 0.05

**p < 0.01

***p < 0.001

## Discussion

This study investigates the relationship between age at sexual debut and pregnancy termination among sexually active young women in 23 SSA countries. The findings indicate that 11.00% of these women experienced pregnancy termination, with the prevalence ranging from 26.85% in Côte d'Ivoire (2012) to 6.55% in Comoros (2012). The prevalence of early sexual debut was 32.93%, with Ethiopia showing the highest rate at 56.50% (2016) and Comoros the lowest at 5.23% (2012).

The study also found that young women who initiated sexual activity before age 16 had a higher likelihood of pregnancy termination compared to those whose debut was at 16 or older. Additional risk factors included age, partner's education, employment, media exposure, and family planning knowledge, while protective factors included place of residence, wealth, parity, and unmet need for contraception.

This study's results on the prevalence of pregnancy termination are consistent with a study conducted by Schwartz et al. [33] among female sex workers in Cote d'Ivoire, which reported that about two-thirds of the respondents between the age of 20–24 years had engaged in pregnancy termination at one point or the other, while more than half had multiple pregnancy terminations prior to when the study was conducted. In the same vein, another study conducted by Bell et al. [34] shows that the younger female respondents in Cote d'Ivoire were more likely to report a higher prevalence of pregnancy termination in the country compared to other age groups. The disaggregation between family planning services and other sexual and reproductive services has been linked to the high prevalence of pregnancy termination among young women in the country [33], which is not the same in some SSA countries like South Africa [35] and Kenya [36].

A higher prevalence of early sexual debut (56.50%) was reported in Ethiopia compared to other SSA countries included in the study. This result is higher than the early sexual debut prevalence of 20.20% in a study conducted among young women in Nigeria [37]. However, a study conducted among young girls in Ethiopia established a higher prevalence of early sexual debut (19%) in the country, which is consistent with this study's result, although the prevalence reported was lower than the prevalence reported in this study [38]. This may be because of some cultural belief within the country or other factors that encourage early marriage, which could be linked to early sexual debut in many cases [6].

Age disparity in sexual experience is a significant risk factor for pregnancy termination. While access to contraceptive services is often highlighted as a solution to reduce pregnancy termination among young women, understanding the role of early sexual debut is equally crucial. Expanding interventions to address early sexual initiation could help prevent pregnancy termination among sexually active young women in SSA [33, 34].

This study reveals that young women in SSA who had an early sexual debut had higher odds of pregnancy termination. Similar findings were observed in Congo and Uganda [39, 40], possibly due to less awareness of sexual and reproductive health services [41].

Young women whose partners had secondary education or higher were found to have higher odds of pregnancy termination. This could be due to the partner's reluctance to start a family, and their education may provide them with the knowledge and persuasive ability to influence the decision to terminate the pregnancy. Previous studies have also documented that partners often play a significant role in pregnancy terminations, highlighting their influence in such decisions [42, 43].

The results showed a higher likelihood of pregnancy termination among young women currently working and those with knowledge of family planning in SSA. This is because early labour force participation requires a lot of dedication and time to carry out assigned tasks, and keeping pregnancy at this stage may be detrimental to their career progression [44–46]. To further buttress this, beyond career concerns, factors such as financial instability, social pressure, and lack of support systems may be the push factor to labour force participation. Young women working early may feel unprepared for the dual responsibilities of career and parenthood, leading to decisions favouring pregnancy termination. In the same vein, young women with knowledge of family planning would have been informed about other sexual and reproductive services available, including abortion services, hence deciding to opt for abortion services in case of family planning use failure or non-adherence to prescribed family planning regimens [47]. These findings have been reported previously in other studies conducted in SSA [29, 48].

The present study further showed that sexually active young women residing in rural areas in SSA had lower odds of having pregnancy termination. Young women residing in the rural areas in SSA might have limited access to pregnancy termination services, which are more prominent in the urban areas in SSA, despite the restrictions on engaging in pregnancy termination except to save the life of the mother in many countries in SSA, frequent pregnancy terminations have been reported in Urban areas in SSA [7, 49]. The unavailability of pregnancy termination services in SSA rural areas is the possible explanation for the result reported in this study, and this study’s result is in line with a study conducted by Ibisomi and Odimegwu [48] in SSA and another study conducted in Malawi by [50] who concluded that respondents in rural areas of Burkina Faso, Gabon, Ghana and Mozambique were more likely to report lower odds of pregnancy termination compared to those residing in urban areas in SSA.

### Strengths and limitations

This study utilised reliable data from a well representative large sample size from 23 countries in SSA, making it possible to generalise the findings to sexually active young women in SSA. Nevertheless, the results reported in this study cannot draw causal interpretations because of the cross-sectional nature of the surveys utilised. Also, there are different legally acceptable definitions of age at sexual debut from different countries included in this study. Such a late sexual debut of 16 years and above may be the appropriate legal age for some countries. In the same vein, pregnancy termination reported by the respondents in this study could be underreported due to stigma or fear, and because it’s self-reported, it could be prone to recall bias that could lead to either under or over-estimation. Also, note that the definition of pregnancy termination by DHS includes stillbirths and miscarriages that the respondents did not intend. Lastly, the surveys included in this study were from different countries in SSA and were conducted in different years, so comparing the findings between these countries may cause bias.

### Policy and public health implications

This study's findings emphasise the need for policy reforms in SSA to help young women with early sexual debut make informed decisions while reducing the rate of pregnancy termination. The policy should focus on providing comprehensive sexual and reproductive health education tailored to this group. Strategies may include awareness campaigns about the connection between early sexual debut and pregnancy termination while promoting informed, correct and consistent use of modern contraceptives. The goal is to ensure young women are empowered to make well-informed choices regarding their sexual health. By implementing these strategies, the burden of adverse public health outcomes can be minimised.

## Conclusion and recommendations

The age at sexual debut among sexually active young women in SSA is significantly associated with pregnancy termination. Specifically, those who had their first sexual experience before age 16 are more likely to engage in pregnancy termination compared to those who had their debut at age 16 or older. Other risk factors linked to pregnancy termination in this group include the respondent's age, partner’s level of education, place of residence, work status, mass media exposure, and knowledge of family planning. These findings highlight the need to integrate such information into existing policies and public health interventions across SSA to help prevent unwanted or unintended pregnancies, which often result in pregnancy termination.

In addition to previous recommendations for providing adequate access to family planning services and counselling, this study suggests that governmental and non-governmental organisations should promote delayed sexual debut among sexually active young women in SSA. This could be achieved through targeted campaigns and incentives, which would accelerate progress toward SDG 3.7. SDG 3.7 aims to ensure universal health coverage, particularly concerning sexual and reproductive health education and information, with the goal of integrating these strategies into national policies by 2030.

Moreover, further qualitative research is necessary to explore the relationship between early sexual debut and adverse reproductive outcomes, such as miscarriage, stillbirth, and induced abortion. Such research would also help validate data from the DHS, offering deeper insights into the causes and consequences of early sexual debut in the region.

## Data Availability

The datasets utilised in this study can be accessed after request and approval via https://dhsprogram.com/data/available-datasets.cfm.
